# Effect of P53 nuclear localization mediated by G3BP1 on ferroptosis in acute liver failure

**DOI:** 10.1007/s10495-023-01856-y

**Published:** 2023-05-27

**Authors:** Wenyuan Li, Wei Li, Xun Li, Luwen Wang, Yao Wang

**Affiliations:** 1 Department of Anesthesiology, Renmin Hospital of Wuhan University, Wuhan, China; 2 Department of Infectious Diseases, Renmin Hospital of Wuhan University, Wuhan, China

**Keywords:** Acute liver failure, Ferroptosis, G3BP1, P53, Nuclear localization

## Abstract

This study investigated whether G3BP1 could regulate ferroptosis in hepatocytes during ALF by affecting the entry of P53 into the nucleus. Promoting G3BP1 expression could inhibit P53 entry by binding to the nuclear localization sequence of P53. The inhibition of SLC7A11 transcription was weakened after blocking of P53 binding to the promoter region of the SLC7A11 gene. The SLC7A11-GSH-GPX4 antiferroptotic pathway was subsequently activated, and the level of ferroptosis in ALF hepatocytes was inhibited.

## Introduction

Acute liver failure (ALF) is a severe and critical condition of liver synthesis, detoxification, and metabolic dysfunction caused by various factors. Its pathological manifestations are severe degeneration and necrosis of a large number of hepatocytes [1]. ALF patients have sudden onset, complicated lesions, intractable treatment and a high fatality rate. Although a set of comprehensive treatment methods have been developed after years of efforts, no breakthrough has been made thus far, and there is still a lack of effective means to prevent liver cell death in ALF [2]. Finding new therapeutic targets to prevent hepatocyte death is critical for alleviating ALF.

Ferroptosis is an important mechanism of hepatocyte death in the pathogenesis of ALF [3]. Ferroptosis in hepatocytes can be effectively prevented by inhibiting reactive oxygen species (ROS) and lipid peroxides [3]. Studies have shown that cystine/glutamate transporter solute carrier family 7 member 11 (SLC7A11) induces glutathione (GSH) synthesis by promoting cystine uptake. Moreover, GSH is catalyzed by the phospholipid hydroperoxide glutathione peroxidase 4 (GPX4) to oxidize and remove lipid peroxides, thereby effectively inhibiting ferroptosis in cells [4, 5]. Therefore, the SLC7A11-GSH-GPX4 axis is a key pathway that protects against ferroptosis in cells, and how to activate this pathway is the focus of this study.

The tumor antigen p53 enters the nucleus via nuclear translocation and inhibits SLC7A11 transcription by binding to the SLC7A11 DNA promoter region, thereby suppressing SLC7A11-GSH-GPX4 axis-mediated ferroptosis [6, 7]. If the nuclear localization of P53 can be inhibited, the transcription of SLC7A11 can be effectively promoted. Previous studies have shown that Ras-GTPase activating protein-SH3-domain-binding protein 1 (G3BP1) binds to p53 and regulates the distribution of p53 between the nucleus and cytoplasm [8]. However, the study did not specify which amino acid sequence of G3BP1 could interact with p53.

The present study further verified the effect of the SLC7A11-GSH-GPX4 axis and G3BP1 on ferroptosis in hepatocytes regulated by the nuclear localization of P53 in the context of ALF. This study clarified the specific molecular mechanism by which G3BP1 regulates the nuclear localization of P53. An in-depth understanding of the relevant mechanism of ALF hepatocyte ferroptosis provides a scientific basis for understanding the pathogenesis and developing treatment strategies for ALF, which has potential clinical value.

## Materials and methods

### Reagents

The normal liver cell line L02 was purchased from the China Center for Type Culture Collection (Wuhan University). Dulbecco’s modified Eagle’s medium (DMEM, Cat. No. 11,995,065) and fetal bovine serum (FBS, Cat. No. 12,483,020) were purchased from Gibco (Grand Island, USA). LPS (Cat. No. L4391), TNF-α (Cat. No. H8916) and D-galactosamine (D-Gal) (Cat. No. G0500) were purchased from Sigma (Grand Island, USA). A bicinchoninic acid (BCA) protein quantification kit (Cat. No. P0012S), cell/tissue protein extraction kit (Cat. No. P0028), cell counting kit-8 (CCK8) kit (Cat. No. C0037), lactate dehydrogenase (LDH) kit (Cat. No. C0016), malondialdehyde (MDA) kit (Cat. No. S0131S), GSH (Cat. No. S0053) kit and ROS (Cat. No. S0033S) kit were purchased from Beyotime (Shanghai, China). A human/mouse p53 enzyme-linked immunosorbent assay (ELISA) kit (Cat. No. ab171571 and ab224878) and iron ion detection kit (Cat. No. ab83366) were purchased from Abcam (Cambridge, UK). Rabbit anti-mouse/human G3BP1 (Cat. No. 13057-2-AP), SLC7A11 (Cat. No. 26864-1-AP) and GPX4 (Cat. No. 67763-1-Ig) primary antibodies were purchased from ProteinTech (Wuhan, China). Goat anti‑rabbit secondary antibodies (IRD ye800CW; Cat. No. 926‑32,211) was purchased from LI‑COR (Lincoln, USA). Rabbit anti‑goat secondary antibodies fluorescently labeled with Cy3 (Cat. No. BA1031 and BA1032) and FITC (Cat. No. BA1101 and BA1105) were purchased from Boster (Wuhan, China). RNAiso Plus (Cat. No. 9108Q), PrimeScript™ RT reagent (Cat. No. RR047Q), and SYBR Premix Ex Taq (Cat. No. RR420A) kits were purchased from Takara (Dalian, China).

### Animal culture and intervention

According to a previous method [9], 30 specific pathogen-free (SPF) male C57BL/6 mice (18–22 g) (aged 8–10 weeks (w)) were obtained from Hubei Provincial Center for Disease Control and Prevention (Wuhan, China). All animal experimental procedures were evaluated and approved by the Laboratory Animal Care and Committee of Renmin Hospital of Wuhan University (WDRM20181018) and followed institutional and ARRIVE guidelines. The mice were randomly divided into the normal group (n = 10), model group (n = 20) and adeno-associated virus (AVV)-G3BP1 intervention group (n = 10). All mice were kept for 7 days (d) to acclimate. The expression of G3BP1 was specifically upregulated in hepatocytes by using an AAV carrying the G3BP1 promoter (Hanbio Biotechnology Co., Ltd.). The AVV-G3BP1 intervention group was injected with the AVV into the caudal vein 1 w before modeling. The model group and the AVV-G3BP1 group were injected with LPS (100 µg/kg) and D-gal (400 mg/kg) [10]. The normal group received an equal amount of saline. At 24 h (h) after modeling, the mice were sacrificed by cervical dislocation. Liver tissue and blood samples were collected for follow-up experiments.

### Cell culture and intervention

According to a previous method [11], the L02 cell line was cultured in DMEM complete culture medium containing 10% FBS. The incubator was set at 37°C and 5% CO_2_. Cells in the logarithmic growth stage were used for subsequent experiments. A G3BP1 overexpression plasmid was constructed and transfected into the LV-G3BP1 group and LV-G3BP1 + Erastin group by a lentivirus (LV)-coated plasmid. After 24 h, the erastin group and LV-G3BP1 + erastin group were treated with 4 mmol/L erastin. After 48 h, cells in the model group, LV-G3BP1 group, erastin group and LV-G3BP1 + erastin group were treated with TNF-α (50 ng/ml) plus D-Gal (70 mmol/ml) [12]. In the siRNA-G3BP1 group and p53-nuclear localization sequences (NLS)^−/−^ group, G3BP1 knockdown siRNA and P53 NLS mutant plasmid were administered to the cells separately or in combination. After 48 h, the cells were treated with TNF-α plus D-Gal. The sequence for G3BP1 knockdown was 5’-CCAAGAUGAGGUCUUU GGUGGGUUUUU-3’ (RIBOBIO, Guangzhou, China).

### Blood biochemistry, liver pathology and hepatocyte mortality assays

The serum in each group was isolated, and the levels of alanine aminotransferase (ALT), aspartate aminotransferase (AST) and total bilirubin (TBil) in the peripheral venous blood of mice were examined by an automatic biochemical analyzer. Hematoxylin-eosin (HE) staining was used to detect histopathological changes in the liver. Liver tissues were fixed with formaldehyde and embedded in paraffin. The wax block was cut into 4 μm-thick slices. The slices were successively washed in xylene, anhydrous ethanol, and distilled water. Hematoxylin staining was performed for 5 min (min). After being rinsed with tap water, the slices were placed in eosin dye solution for 2 min. The slices were then placed into anhydrous ethanol and xylene in turn, dehydrated until transparent, dried and sealed. TdT-mediated dUTP nick end labeling (TUNEL) staining was used to detect the apoptosis index of hepatocytes. The sections were stained according to the procedure of the TUNEL assay kit and photographed for analysis with a fluorescence microscope. Three specimens were used for cell counting using as described previously. The conventional labeling index (LI) was used: LI = number of positive cells per field of labeling/number of all cells in the field of labeling. Three visual fields were selected randomly, and the apoptosis index (AI) of each group was equal to the average value of the landmark index of various visual fields [13].

### Cell activity, LDH, ROS, MDA, GSH and Fe^2+^ assays

As previously described [10], the CCK8 method was used to measure cell activity in each group. The cells were inoculated in 96-well plates and treated as indicated: cell activity (%) = experimental group optical density (OD) value/control group OD value ×100%. The cells or liver tissues in each group were treated and homogenized. After the supernatant was collected, LDH, ROS, MDA, GSH and Fe^2+^ levels were detected according to the instructions of the test kits.

### G3BP1 and SLC7A11 mRNA analysis by RT‒PCR

As previously described [14], total RNA was extracted from each group of cells or animals by an RNAiso Plus kit. The extracted RNA was reverse-transcribed into cDNA by PrimeScript™ RT reagent. The reverse transcription program was 37 °C for 15 min and at 85 °C for 5 s. By using the SYBR Premix Ex Taq kit, cDNA was subjected to quantitative polymerase chain reaction using an RT‒PCR instrument. The PCR program was set at 95 °C for 5 s, followed by 95 °C for 5 s and 60 °C for 34 s for a total of 40 cycles. The relative gene expression levels of G3BP1 and SLC7A11 were calculated by the 2^−ΔΔCT^ method. The PCR primers were designed and constructed by Tsingke (Beijing, China), and the specific sequences are shown in Table 1.

**Table 1:** Primers for RT‒PCR

Genes	Forward (5′-3′)	Reverse (5′-3′)
G3BP1(mouse)	TTGGAGGAGCATTTAGAGGAGC	TCTTGAATGTCGGACACAGGT
G3BP1(human)	AAGAGTGCGAGAACAACGAA	TGGTGACTGTCAGGGTGTCT
SLC7A11 (mouse)	CTTTGTTGCCCTCTCCTGCTTC	CAGAGGAGTGTGCTTGTGGACA
SLC7A11 (human)	ATGCAGTGGCAGTGACCTTT	GGCAACAAAGATCGGAACTG
GAPDH (mouse)	ACCACAGTCCATGCCATCAC	TCCACCACCCTGTTGCTGTA
GAPDH (human)	ACCACAGTCCATGCCATCAC	TCCACCACCCTGTTGCTGTA

### G3BP1, GPX4, SLC7A11 and P53 protein analysis

Nuclear and cytoplasmic protein extraction kits were used to extract proteins from cells and tissues in each group according to the kit instructions. An ELISA kit was used to measure the P53 protein levels. The total protein level was the sum of the levels of nuclear and cytoplasmic proteins. The protein levels of G3BP1, SLC7A11 and GPX4 were determined by western blotting. Cell or animal specimens were collected into centrifuge tubes. Total protein was extracted, and the protein concentration was determined by the BCA method. SDS‒PAGE was performed, and the skim milk powder was sealed after the transfer. Rabbit anti-human/mouse primary anti-G3BP1 (1:1000), GPX4 (1:1000), SLC7A11 (1:1000), and GAPDH (1:1000) antibodies were used. After the membranes were washed, they were incubated with goat anti-rabbit secondary antibodies. Finally, the Odyssey system was used to scan the membranes and analyze the expression levels of each protein.

### Detection and localization of G3BP1 and P53 in hepatocytes by confocal microscopy

The cells in each group were plated in 24-well plates and treated accordingly. After being washed, the cells were fixed, followed by Triton permeation and BSA sealing. Primary antibodies against G3BP1 and P53 were added and incubated overnight. The next day, after the second antibody incubation, DAPI was added. After the samples were washed with PBS, an anti-fluorescence quencher was added, and the tablets were sealed. The expression and localization of G3BP1 and P53 were observed by confocal microscopy.

### Statistical analysis

SPSS 19.0 statistical software was used for statistical analysis of the data, and Prism 8.0 software was used to plot the graphs. All data are expressed as the mean ± standard deviation ($\bar x$ ± s). The t test was used to compare the two groups. P < 0.05 was considered statistically significant.

## Results

### Effects of G3BP1 on ferroptosis, pathology and blood biochemistry in ALF mice

**Fig. 1:**
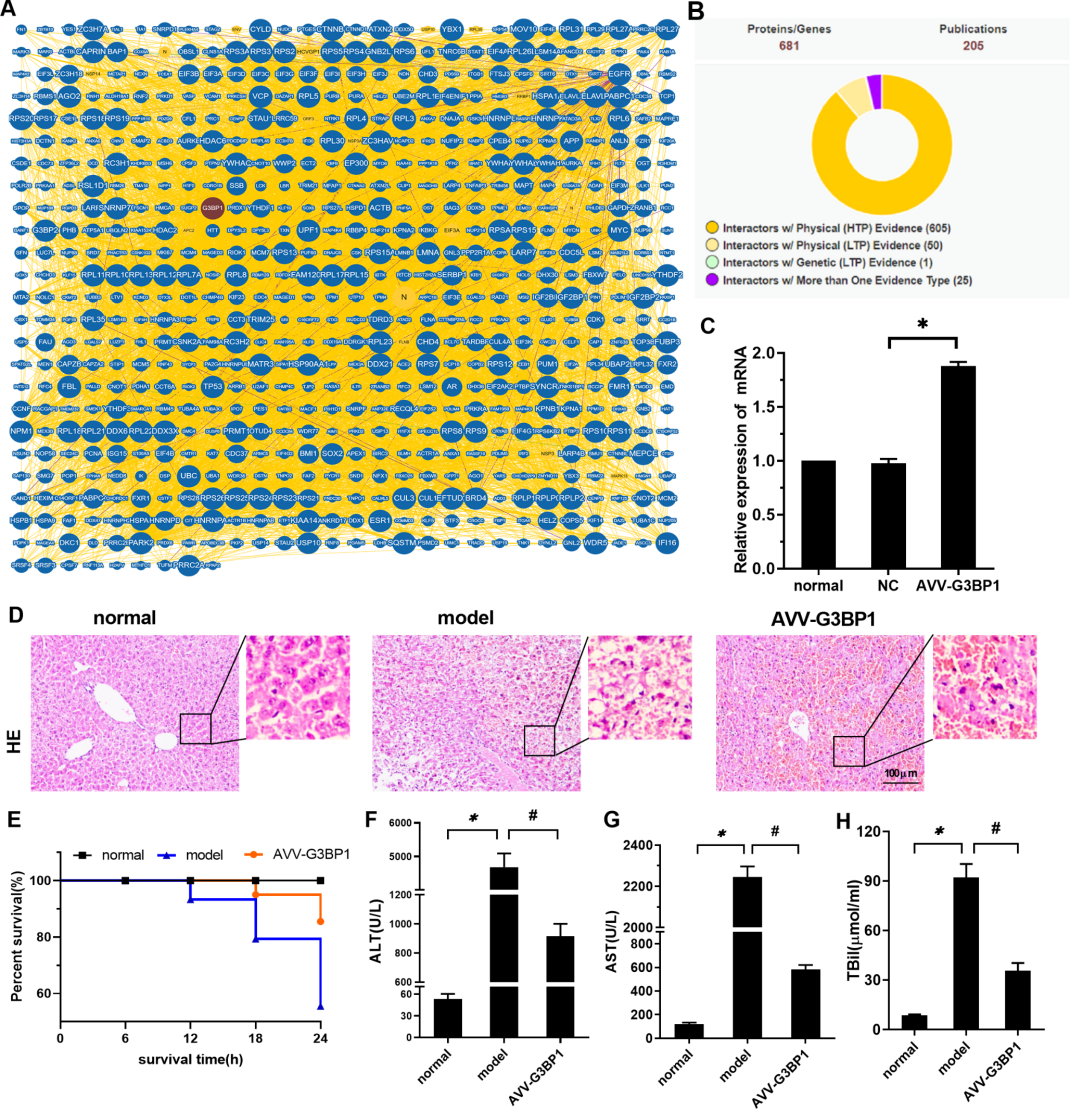
Effects of G3BP1 on ferroptosis, pathology and blood biochemistry in ALF mice. (**A**) The BioGRID database of proteins that interact with G3BP1. (**B**) The results combined the 681 proteins, and 205 publications reported on these interactions. (**C**) G3BP1 mRNA was detected by RT‒PCR. (**E**) The 6 h, 12 h, 18 h, and 24 h survival rates of mice in each group were observed. (**F-H**) The levels of ALT, AST and TBIL in the serum of mice were detected by an automatic biochemical analyzer. N = 10. The data are shown $\bar x$ ± s. * *P < 0.05*, compared with the normal group. # *P < 0.05*, compared with the model group

We first searched the BioGRID database for proteins that interact with G3BP1. As shown in Fig. 1A, the network diagram shows the interaction of these proteins. G3BP1 can interact with TP53 (P53). As shown in Fig. 1B, we combined the 681 proteins, and 205 publications reported on these interactions. To further verify the influence of the interaction between G3BP1 and P53 on ferroptosis during ALF, we first used a mouse model of ALF to overexpress G3BP1 in the liver. As shown in Fig. 1C, compared with that in the normal group, the mRNA expression of G3BP1 in the normal control (NC) group did not change, while that in the AVV-G3BP1 group was increased (P < 0.05). This finding indicated that AVV itself had no effect on the expression of G3BP1. The overexpression vector successfully overexpressed G3BP1 in the liver and was used for subsequent experiments.

As shown in Fig. 1D, hepatocytes in the normal group were arranged neatly with a clear cell structure. In the model group, a large number of hepatocytes were necrotic, and the structure of the hepatic lobule was destroyed. There was obvious inflammatory cell infiltration. Compared with those in the model group, the degree of hepatocyte necrosis and inflammatory infiltration in the AVV-G3BP1 group were significantly reduced. As shown in Fig. 1E, the 24 h survival rate showed that 90% of mice survived in the AVV-G3BP1 group, whereas only 70% survived in the model group. As shown in Fig. 1F H, serum ALT, AST and TBil levels in the model group were increased compared with those in the normal group (P < 0.05). Compared with those in the model group, ALT, AST and TBil levels were decreased in the AVV-G3BP1 group (P < 0.05).

### Effect of G3BP1 on ferroptosis levels in the liver tissue of ALF mice

Oxidative stress may mediate ferroptosis in hepatocytes. Therefore, referring to previous studies [3, 15], we observed apoptosis levels in liver tissues and the changes in the oxidative stress damage-related molecules MDA, GSH and LDH and selected the key molecules of ferroptosis (GPX4, ROS, SLC7A11 and Fe^2+^) as the research objects. Moreover, the mRNA level of SLC7A11 was regulated after P53 entered the nucleus [16]. Therefore, this experiment was conducted to observe whether G3BP1 affected P53 entry into the nucleus and the transcription of SLC7A11 and its mediation of ferroptosis in hepatocytes.

**Fig. 2:**
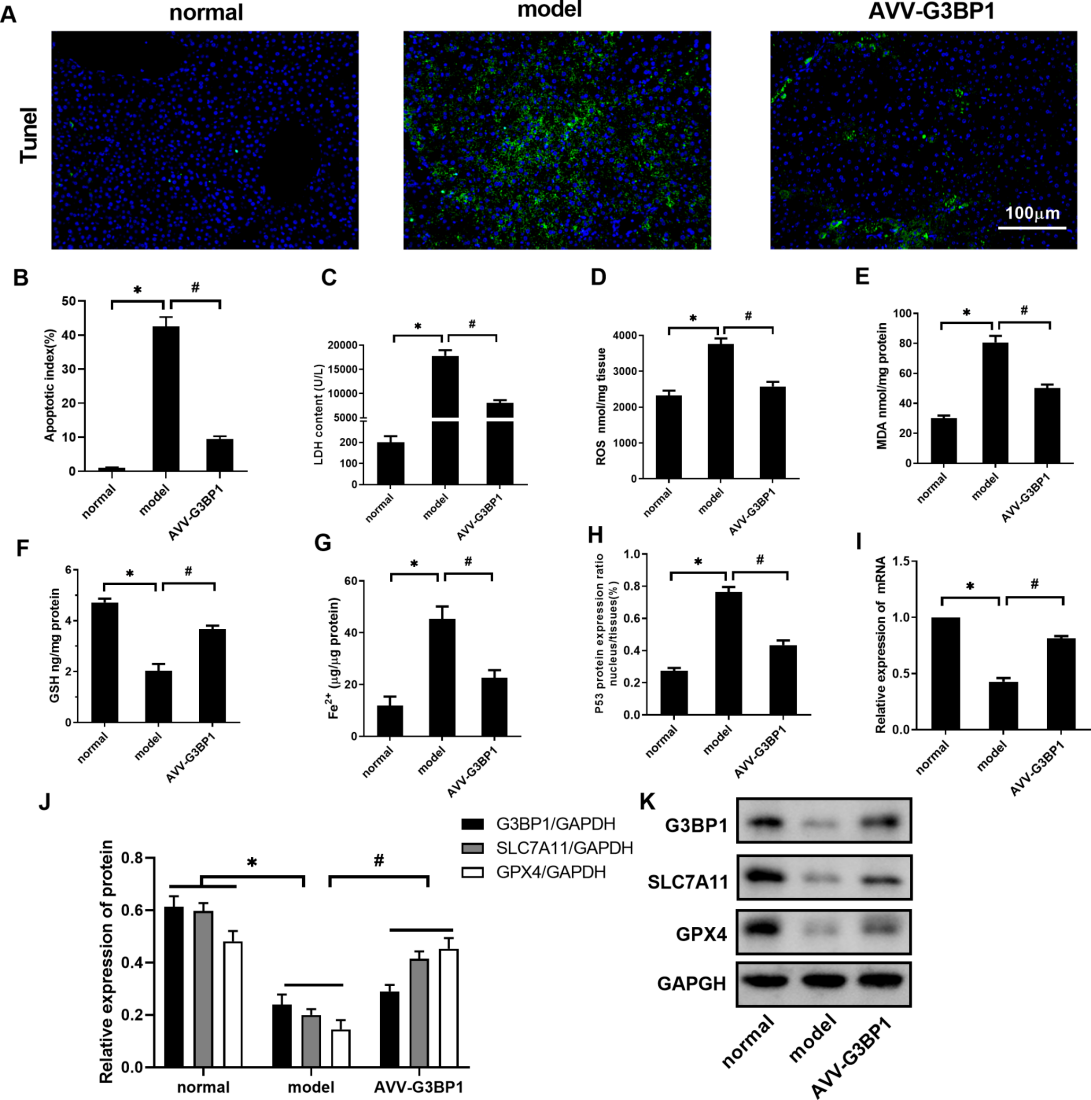
Effect of G3BP1 on ferroptosis levels in ALF mouse liver tissue. (**A-B**) TUNEL staining was used to detect the level of apoptosis in liver tissue. (**C-G**) The levels of LDH, ROS, MDA, GSH, and Fe^2+^ were detected by test kits. (**H**) P53 protein expression was detected by an ELISA kit. (**I**) SLC7A11 mRNA was detected by RT‒PCR. (**J-K**) The protein levels of G3BP1, SLC7A11 and GPX4 were determined by western blotting. N = 10. The data are shown $\bar x$ ± s. * *P < 0.05*, compared with the normal group. # *P < 0.05*, compared with the model group

**Fig. 3:**
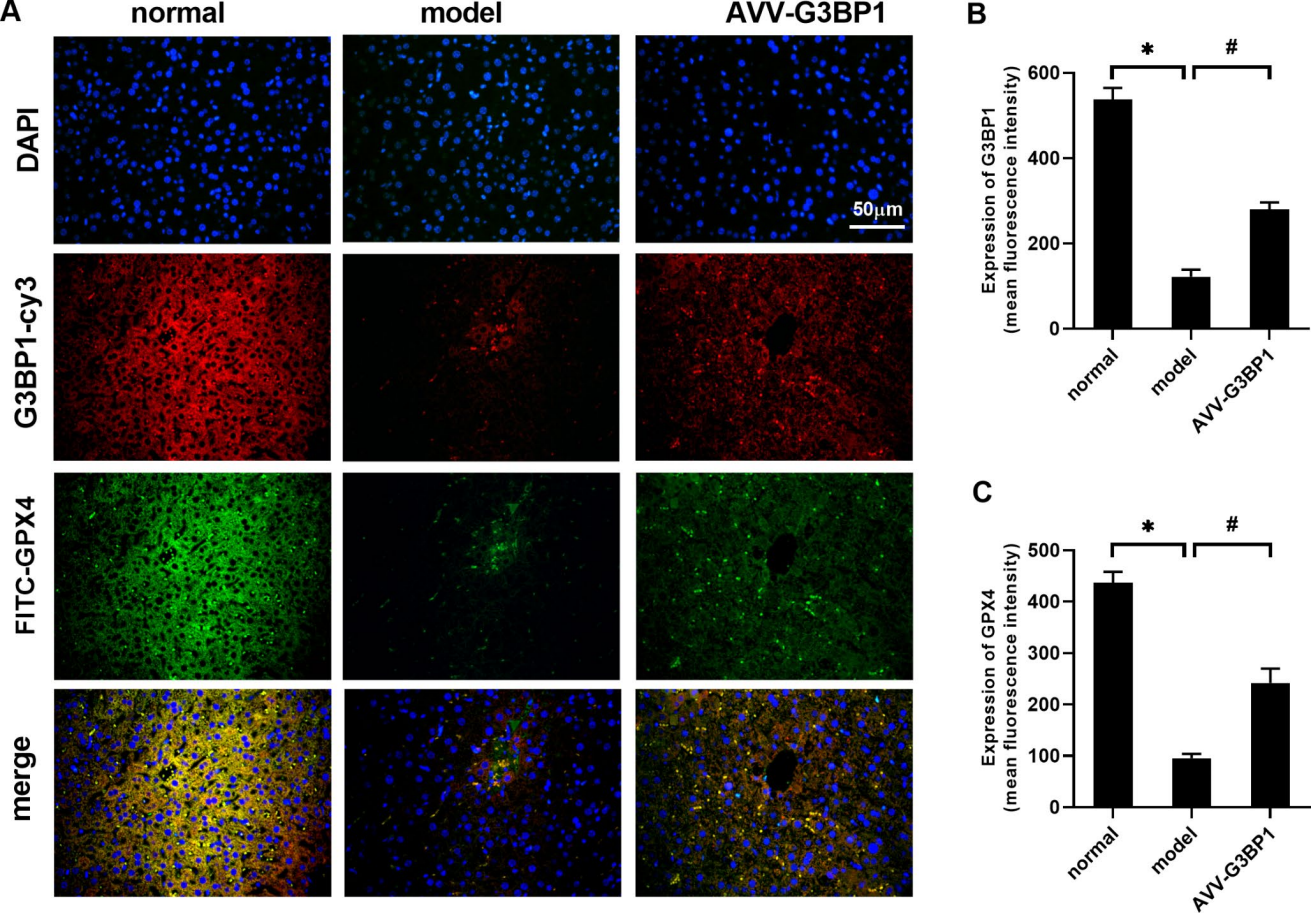
(**A-C**) The protein levels of G3BP1 and GPX4 in liver tissue were observed by fluorescence staining. N = 3. The data are shown $\bar x$ ± s. * *P < 0.05*, compared with the normal group. # *P < 0.05*, compared with the model group

As shown in Fig. 2A and G, the levels of the apoptotic index, LDH, ROS, MDA, and Fe^2+^ were increased in the model group compared with the normal group (P < 0.05), and GSH levels were decreased (P < 0.05). Compared with those in the model group, the levels of the apoptotic index, LDH, ROS, MDA, and Fe^2+^ were decreased in the AVV-G3BP1 group (P < 0.05), and GSH levels were increased (P < 0.05). The ratio of nuclear P53 to total P53 protein (P53^N/T^) was higher in the model group than in the normal group (P < 0.05). This finding indicated that the P53 protein was transferred into the nucleus during ALF. Compared with that in the model group, the P53^N/T^ level in the AVV-G3BP1 group was decreased (P < 0.05), which indicated that G3BP1 overexpression could inhibit P53 nuclear translocation. As shown in Fig. 2I K, we further analyzed the expression of SLC7A11 mRNA, G3BP1, and marker proteins of ferroptosis (GPX4 and SLC7A11). Compared with the normal group, SLC7A11 mRNA, G3BP1, GPX4 and SLC7A11 protein levels were decreased in the model group (P < 0.05). Compared with those in the model group, SLC7A11 mRNA and G3BP1, GPX4 and SLC7A11 protein levels were increased in the AVV-G3BP1 group (P < 0.05). As shown in Fig. 3A C, fluorescence staining of liver tissue showed that compared with those in the normal group, the protein levels of G3BP1 and GPX4 in the model group were decreased (P < 0.05). Compared with those in the model group, the protein levels of G3BP1 and GPX4 were increased in the AVV-G3BP1 group (P < 0.05).

### Effect of G3BP1 on ferroptosis in hepatocytes

**Fig. 4:**
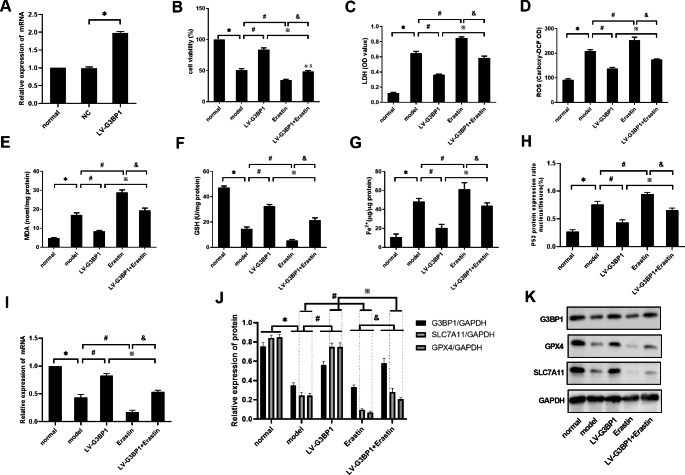
Effect of G3BP1 on ferroptosis in hepatocytes. (**A**) G3BP1 mRNA was detected by RT‒PCR. (**B-G**) The levels of cell viability, LDH, ROS, MDA, GSH, and Fe^2+^ were detected by test kits. (**H**) P53 protein expression was detected by an ELISA kit. (**I**) SLC7A11 mRNA was detected by RT‒PCR. (**J-K**) The protein levels of G3BP1, SLC7A11 and GPX4 were determined by western blotting. N = 3. The data are shown $\bar x$ ± s. * *P < 0.05*, compared with the normal group. # *P < 0.05*, compared with the model group. ※ *P < 0.05*, compared with the LV-G3BP1 group. & *P < 0.05*, compared with the erastin group

In vitro, G3BP1 was overexpressed in the hepatocytes in the model group to verify the effect of G3BP1 on ferroptosis-related molecules. The effect of a ferroptosis agonist on the occurrence of ferroptosis was further observed in the model group of cells overexpressing G3BP1. As shown in Fig. 4A, the effect of lentiviral vector-mediated overexpression of G3BP1 on L02 cells was first verified. Compared with that in the normal group, the G3BP1 mRNA level in the NC group was not significantly changed but was increased in the LV-G3BP1 group (P < 0.05). As shown in Fig. 4B and I, the levels of cell activity, GSH and SLC7A11 mRNA were significantly decreased in the model group compared with the normal group (P < 0.05), and the levels of LDH, ROS, MDH, Fe^2+^ and P53^N/T^ were increased (P < 0.05). Compared with those in the model group, the levels of cell activity, GSH and SLC7A11 mRNA were significantly increased in the LV-G3BP1 group (P < 0.05). The levels of LDH, ROS, MDH, Fe^2+^ and P53^N/T^ were significantly decreased (P < 0.05). The levels of cell activity, GSH and SLC7A11 mRNA were further decreased in the erastin group (P < 0.05), and the levels of LDH, ROS, MDH, Fe^2+^, and P53^N/T^ were further increased (P < 0.05) compared with those in the model group.

Compared with those in the LV-G3BP1 group, the levels of cell activity, GSH and SLC7A11 mRNA were significantly decreased in the LV-G3BP1 + Erastin group (P < 0.05). The levels of LDH, ROS, MDH, Fe^2+^ and P53^N/T^ were significantly increased (P < 0.05). Compared with those in the Erastin group, the levels of cell activity, GSH and SLC7A11 mRNA were significantly increased in the LV-G3BP1 + Erastin group (P < 0.05). The levels of LDH, ROS, MDH, Fe^2+^ and P53^N/T^ were significantly decreased (P < 0.05).

As shown in Fig. 4J K, compared with those in the normal group, the protein levels of G3BP1, GPX4 and SLC7A11 in the model group were significantly decreased (P < 0.05). Compared with those in the model group, the protein levels of G3BP1, GPX4 and SLC7A11 in the LV-G3BP1 group were significantly increased (P < 0.05), and the levels of GPX4 and SLC7A11 in the erastin group were further decreased (P < 0.05). Compared with those in the LV-G3BP1 intervention group, GPX4 and SLC7A11 protein levels were decreased in the LV-G3BP1 + Erastin group (P < 0.05). Compared with those in the Erastin group, the protein levels of G3BP1, GPX4 and SLC7A11 were increased in the LV-G3BP1 + Erastin group (P < 0.05).

### G3BP1 affects ferroptosis by regulating the SLC7A11-GSH-GPX4 axis through the P53 NLS

**Fig. 5:**
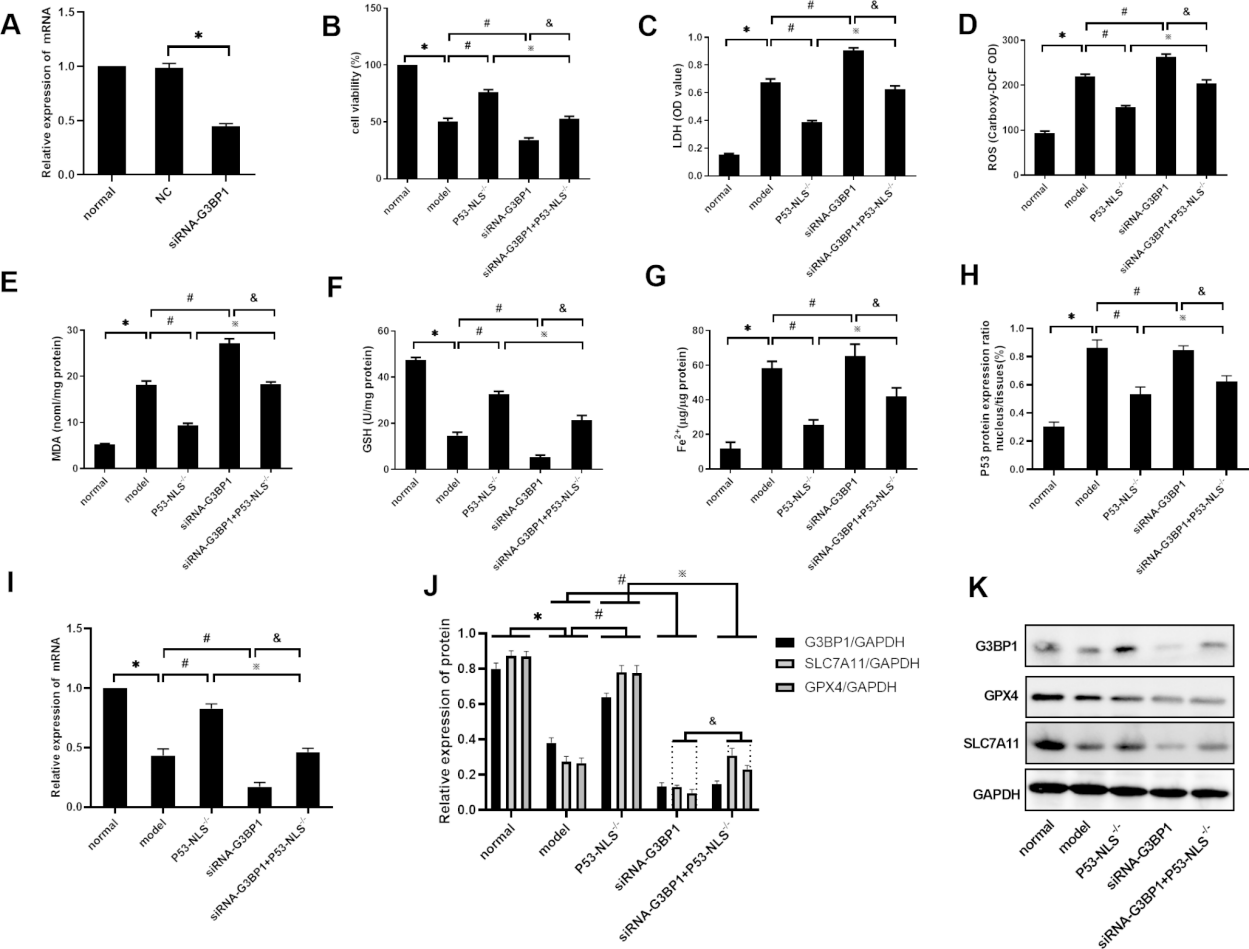
G3BP1 affects ferroptosis by regulating the SLC7A11-GSH-GPX4 axis through the P53 NLS. (**A**) G3BP1 mRNA was detected by RT‒PCR. (**B-G**) The levels of cell viability, LDH, ROS, MDA, GSH, and Fe^2+^ were detected by test kits. (**H**) P53 protein expression was detected by an ELISA kit. (**I**) SLC7A11 mRNA was detected by RT‒PCR. (**J-K**) The protein levels of G3BP1, SLC7A11 and GPX4 were determined by western blotting. N = 3. The data are shown $\bar x$ ± s. * *P < 0.05*, compared with the normal group. # *P < 0.05*, compared with the model group. ※ *P < 0.05*, compared with the P53-NLS^−/−^ group. & *P < 0.05*, compared with the siRNA-G3BP1 group

The NLS sequence of P53 was subsequently mutated in model group cells. Ferroptosis was observed after P53 entry into the nucleus was inhibited. Based on the mutant P53 NLS sequence, the expression of G3BP1 was inhibited to determine whether the promotion of ferroptosis after mutation of the P53 NLS sequence could be neutralized. As shown in Fig. 5A, the effect of siRNA-mediated knockdown on G3BP1 in L02 cells was first verified. Compared with that in the normal group, the G3BP1 mRNA level in the NC group was not significantly changed, but the G3BP1 mRNA level in the siRNA-G3BP1 group was decreased (P < 0.05). As shown in Fig. 5B and I, the levels of cell activity, GSH and SLC7A11 mRNA were significantly decreased in the model group compared with the normal group (P < 0.05), and the levels of LDH, ROS, MDH, Fe^2+^ and P53^N/T^ were significantly increased (P < 0.05). Compared with those in the model group, the levels of cell activity, GSH and SLC7A11 mRNA in the P53-NLS^−/−^ group were significantly increased (P < 0.05), and the levels of LDH, ROS, MDH, Fe^2+^ and P53^N/T^ were significantly decreased (P < 0.05). Compared with those in the model group, the levels of cell activity, GSH and SLC7A11 mRNA were further decreased in the siRNA-G3BP1 group (P < 0.05), and the levels of LDH, ROS, MDH, Fe^2+^, and P53^N/T^ were further increased (P < 0.05).

Compared with those in the P53-NLS^−/−^ group, the levels of cell activity, GSH and SLC7A11 mRNA in the siRNA-G3BP1 + P53-NLS^−/−^ group were significantly decreased (P < 0.05), and the levels of LDH, ROS, MDH, Fe^2+^ and P53^N/T^ were significantly increased (P < 0.05). Compared with those in the siRNA-G3BP1 group, the levels of cell activity, GSH and SLC7A11 mRNA in the siRNA-G3BP1 + P53-NLS^−/−^ group were significantly increased (P < 0.05), and the levels of LDH, ROS, MDH, Fe^2+^ and P53^N/T^ were significantly decreased (P < 0.05).

As shown in Fig. 5J K, compared with those in the normal group, G3BP1, GPX4 and SLC7A11 protein levels in the model group were significantly decreased (P < 0.05). Compared with those in the model group, GPX4 and SLC7A11 protein levels in the P53-NLS^−/−^ group were significantly increased (P < 0.05), and G3BP1 protein levels were not significantly changed. The protein levels of G3BP1, GPX4 and SLC7A11 in the siRNA-G3BP1 group were further decreased (P < 0.05). Compared with those in the P53-NLS^−/−^ group, the protein levels of G3BP1, GPX4 and SLC7A11 in the siRNA-G3BP1 + P53-NLS^−/−^ group were decreased (P < 0.05). Compared with those in the siRNA-G3BP1 group, GPX4 and SLC7A11 protein levels in the siRNA-G3BP1 + P53-NLS^−/−^ group were increased (P < 0.05). There was no change in G3BP1 protein levels.

### Domain analysis of the interaction between P53 and G3BP1

**Fig. 6:**
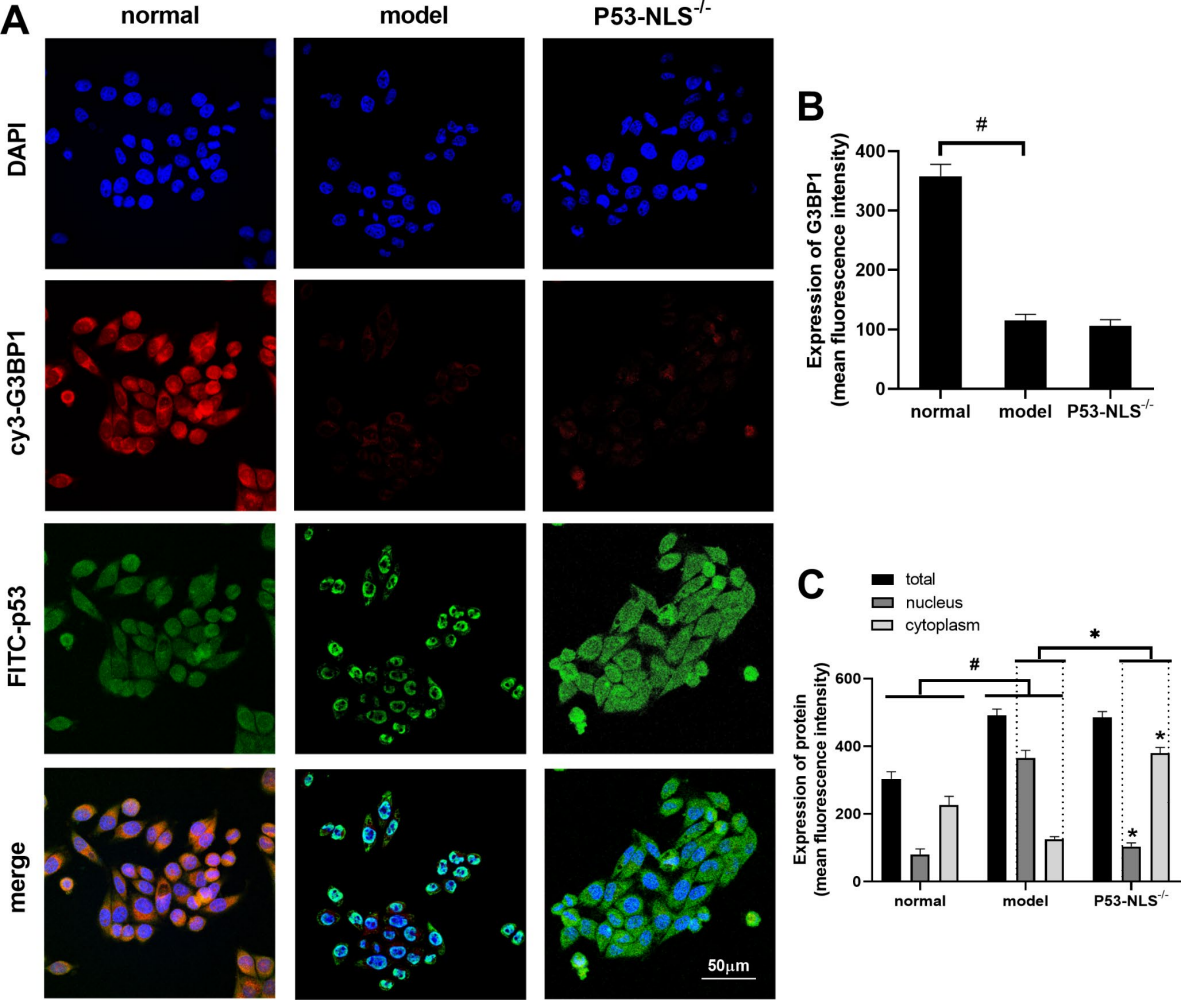
(**A-C**) The protein levels of G3BP1 and P53 in L02 cells were observed by fluorescence staining. N = 3. The data are shown $\bar x$ ± s. * *P < 0.05*, compared with the normal group. # *P < 0.05*, compared with the model group

To more directly observe the location and level of the interaction between P53 and G3BP1 in cells, confocal microscopy was used to observe the fluorescence of P53 and G3BP1. As shown in Fig. 6A and B, G3BP1 exhibited the red fluorescence of cy3 and was mainly expressed in the cytoplasm. Compared with that in the normal group, the expression of G3BP1 in the model group was decreased (P < 0.05). Compared with that in the model group, the expression of G3BP1 in the P53-NLS^−/−^ group did not change. As shown in Fig. 6A C, the green fluorescent protein FITC was used to label P53. In the normal group and p53-NLS^−/−^ group, P53 was mainly expressed in the cytoplasm, while in the model group, P53 was mainly expressed in the nucleus. Compared with those in the normal group, total and nuclear P53 levels in the model group were increased (P < 0.05), and P53 levels in the cytoplasm were decreased (P < 0.05). Compared with those the model group, total P53 levels in the p53-NLS^−/−^ group were unchanged, while cytoplasmic P53 levels were increased (P < 0.05) and nuclear P53 levels ere decreased (P < 0.05). These results indicate that during the inflammatory reaction of hepatocytes, the expression level of G3BP1 was decreased, while the expression level of P53 was increased, and the protein entered the nucleus. However, the mutant P53 NLS sequence did not affect the total protein expression of P53 and G3BP1 but inhibited the entry of P53 into the nucleus and subsequently increased the expression of P53 in the cytoplasm.

**Fig. 7:**
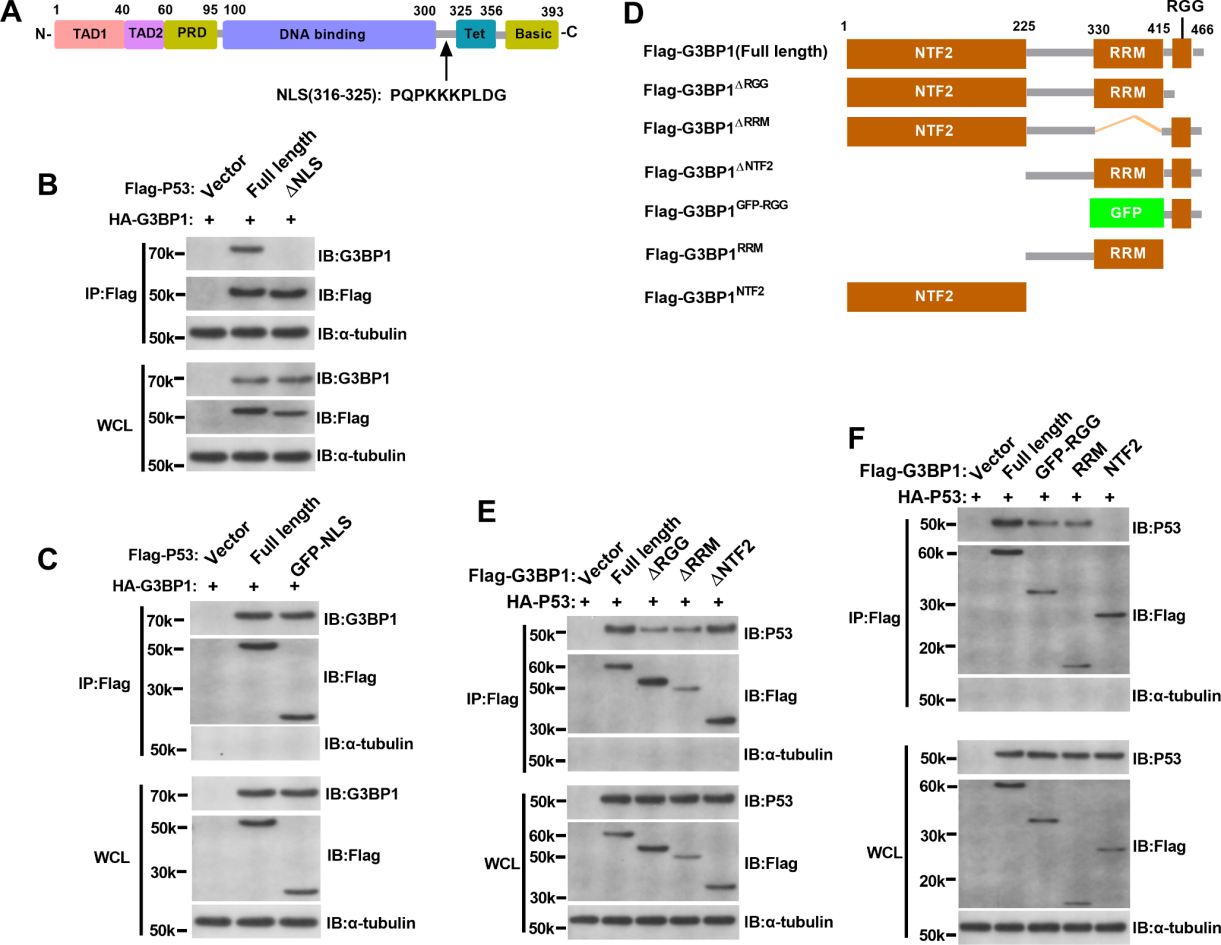
(**A**) Schematic diagram of full-length P53 and the NLS-truncated form. (**B-C**) Analysis of the interaction between the P53 NLS domain and G3BP1. (**D**) Schematic diagram of the full-length and different truncated forms of G3BP1. (**E-F**) Analysis of the interaction between the different domains of G3BP1 and P53

As shown in Fig. 7A, the NLS of P53 is located at amino acid residues 316–325, and we constructed P53 with the following Flag tags: absence of NLS (Flag-P53^ΔNLS^). As shown in Fig. 7B, we simultaneously overexpressed the G3BP1 plasmid with an HA tag and full-length P53 and NLS-truncated granules with a Flag tag in L02 cells. Then, M2 beads were used to immunoprecipitate NLS-truncated bodies. Only full-length P53 could enrich HA-G3BP1, suggesting that the full-length P53 protein could interact with the G3BP1 protein. As shown in Fig. 7C, to more directly analyze the interaction between the two proteins, we incubated purified recombinant G3BP1 protein, full-length P53 containing a Flag label and the recombinant NLS protein. The M2 beads were used to enrich the NLS. Because the NLS motif was too short, we added a GFP tag to its N end in addition to the Flag tag. Similar results were obtained, and both full-length P53 and the NLS were able to enrich recombinant G3BP1 protein, indicating that P53 interacts with G3BP1 through the NLS.

### Domain analysis of the interaction between G3BP1 and P53

Figure 7D shows the full-length and different truncated forms of G3BP1. G3BP1 contains three conserved domains: the nuclear transporter factor 2 (NTF2) domain, RNA-recognition module (RRM) domain and arginine-glycine-glycine (RGG) motif. Based on this structure, we built the following G3BP1 with Flag tags: full length (Flag-G3BP1 (full length)), RGG-null (Flag-G3BP1^ΔRGG^), RRM-null (Flag-G3BP1^ΔRRM^), NTF2-null (Flag-G3BP1^ΔNTF2^), RGG motif (Flag-G3BP1^GFP − RGG^), RRM domain (Flag-G3BP1^RRM^), and NTF2 domain (Flag-G3BP1^NTF2^). Among them, because the RGG motif was too short, we also added a GFP tag to its N-terminus in addition to the Flag tag.

As shown in Fig. 7E, the P53 plasmid with an HA tag was overexpressed in L02 cells at the same time as full-length G3BP1 with a Flag tag and various truncated forms. Then, M2 beads were used to immunoprecipitate the different truncated forms of G3BP1 to determine the specific structural domain of G3BP1 that binds with P53. Full-length G3BP1 lacking NTF2 (ΔNTF2) could enrich HA-P53. However, the absence of RGG (ΔRGG) and RRM (ΔRRM) in G3BP1 significantly reduced the enrichment of HA-P53 Flag tags. This finding suggested that the region that interacts with P53 might be located in the RGG or RRM region of G3BP1. As shown in Fig. 7F, to more directly analyze the interaction between the two proteins, we incubated purified recombinant G3BP1 protein, full-length P53 with a Flag label and recombinant NTF2, RGG and RRM proteins. Similarly, M2 beads are used to enrich NTF2, RGG and RRM. Because the RGG motif is too short, we added a GFP tag to its N-terminus in addition to a Flag tag. Similar results were obtained: full-length G3BP1, RGG and RRM could enrich recombinant P53 protein, while NTF2 could not enrich recombinant P53 protein. This finding further suggested that the regions that interact with P53 might be located in the RGG and RRM regions of G3BP1.

## Discussion

The pathological manifestations of ALF include massive hepatocyte necrosis, inflammatory injury and oxidative stress injury [1]. At present, treatments for hepatocyte death during ALF are still immature [17]. Ferroptosis is a new iron-dependent nonapoptotic cell death mode that was first discovered by Dr. Brent R Stockwell in 2012. Ferroptosis involves the catalysis of iron ions, resulting in redox imbalance in cells that leads to ROS accumulation and cell death [18]. Ferroptosis is characterized by enhanced oxidative stress, intracellular iron ion accumulation, and lipid peroxidation [19].

In recent years, studies have gradually revealed that ferroptosis plays an important role in the occurrence and development of a variety of liver-related diseases. For example, the ferroptosis inhibitor ferrostatin-1 can significantly reduce alcohol-induced hepatocyte death and significantly improve alcohol-induced liver injury in mice [20]. Ferroptosis inhibitors also significantly inhibit hepatocyte death and inflammation-related gene expression in nonalcoholic steatohepatitis (NASH) model mice [21]. Magnesium isoglycyrrhizinate regulates iron transport by upregulating the expression of heme oxygenase 1 (HO-1), promotes the accumulation of Fe^2+^ and lipid peroxides, and induces ferroptosis in hepatic stellate cells (HSCs). Thus, the degree of liver fibrosis can be reduced [22]. However, research on ferroptosis and ALF is still in the initial stage. We confirmed for the first time that hepatocyte ferroptosis occurred in ALF mice induced by LPS/D-Gal. Glycyrrhizin can reduce oxidative stress, inhibit ferroptosis, and alleviate hepatocyte damage by upregulating the Nrf2 signaling pathway [3]. Therefore, ferroptosis is an important mechanism of hepatocyte death during ALF, and inhibiting ferroptosis can effectively alleviate hepatocyte damage. The urgency is that ALF develops rapidly and may progress to hepatic encephalopathy (HE) and can even cause cerebral edema or multiple organ failure. There is no effective treatment for ALF in the clinic. Although liver transplantation can be an effective means, it has not been widely used in the clinic due to the lack of donor livers, postoperative rejection, postoperative ischemia reperfusion injury and other factors. According to the pathogenesis of ALF, timely intervention in hepatocyte ferroptosis is the key to the treatment of ALF.

G3BP1 mainly exists in the cytoplasm, and its C-terminus has RNA binding sites, which participate in mRNA binding and regulate a variety of biological functions, including cell proliferation, invasion and metastasis, apoptosis, differentiation and RNA metabolism [23]. G3BP1 plays an important regulatory role in tumors, viral infection, nervous system diseases and cardiovascular diseases [23]. To identify the target of G3BP1 associated with ferroptosis, we first searched the BioGRID database and showed that P53 interacts with G3BP1. The mouse model of ALF was then used to overexpress G3BP1 in the liver. The results showed that promoting the expression of G3BP1 could reduce the mortality of ALF mice, reduce the degree of liver tissue injury, and alleviate changes in liver function.

Subsequently, we verified whether G3BP1 affected the entry of P53 into the nucleus and interfered with the transcription of SLC7A11 and its effect on ferroptosis in hepatocytes. We found that the level of G3BP1 in the liver was decreased in the model group. The level of ferroptosis and the ratio of nuclear P53 to total P53 protein were increased. The mRNA level of SLC7A11 was decreased. These results indicated that the expression of G3BP1 was decreased during the development of ALF. The intranuclear transfer of P53 occurred during ALF. The transcription level of SLC7A11 in the nucleus was decreased, inducing ferroptosis. After the overexpression of G3BP1 in the liver, the ferroptosis level was decreased, the ratio of nuclear P53 to total P53 protein was decreased, and SLC7A11 mRNA levels were increased. These results suggested that G3BP1 could inhibit the entry of the P53 protein into the nucleus and decrease SLC7A11 transcription and liver cell ferroptosis during ALF.

In vitro, a ferroptosis agonist was administered to hepatocyte injury models overexpressing G3BP1 to further observe the effect of G3BP1 on ferroptosis. We obtained the same results as in vivo. G3BP1 overexpression inhibited the entry of the P53 protein into the nucleus and decreased SLC7A11 transcription and ferroptosis in hepatocytes. Subsequently, we mutated the P53 NLS in the model group and observed ferroptosis in hepatocytes after inhibiting P53 entry into the nucleus. The expression of G3BP1 was inhibited by the mutant P53 NLS sequence. Mutating the P53 NLS sequence caused a decrease in ferroptosis levels. Overexpression of G3BP1 neutralized this promotion of ferroptosis. To more directly observe the site and degree of the interaction between P53 and G3BP1, laser confocal microscopy was used, and during the inflammatory reaction in hepatocytes, the expression level of G3BP1 decreased, while the expression level of P53 increased, and the protein entered the nucleus. However, the mutant P53 NLS sequence did not affect the total protein expression of P53 and G3BP1 but inhibited the entry of P53 into the nucleus and subsequently increased the expression of P53 in the cytoplasm.

Further mechanistic studies were conducted to identify the domain of G3BP1 that interacts with P53. We mutated the P53 NLS and found that only full-length P53 and its NLS could be enriched by G3BP1. Subsequently, we mutated the NTF2, RRM and RGG3 conformal domains of G3BP1. Full-length G3BP1, RGG and RRM could enrich recombinant P53 recombinant protein. This finding indicated that the regions that interact with P53 were located in the RGG and RRM regions of G3BP1. Therefore, G3BP1 could target the nuclear localization sequence in the P53 C-terminus, and this binding could block the entry of P53 into the nucleus, resulting in abnormal retention of p53 in the cytoplasm, which was consistent with previous reports [8].

**Fig. 8:**
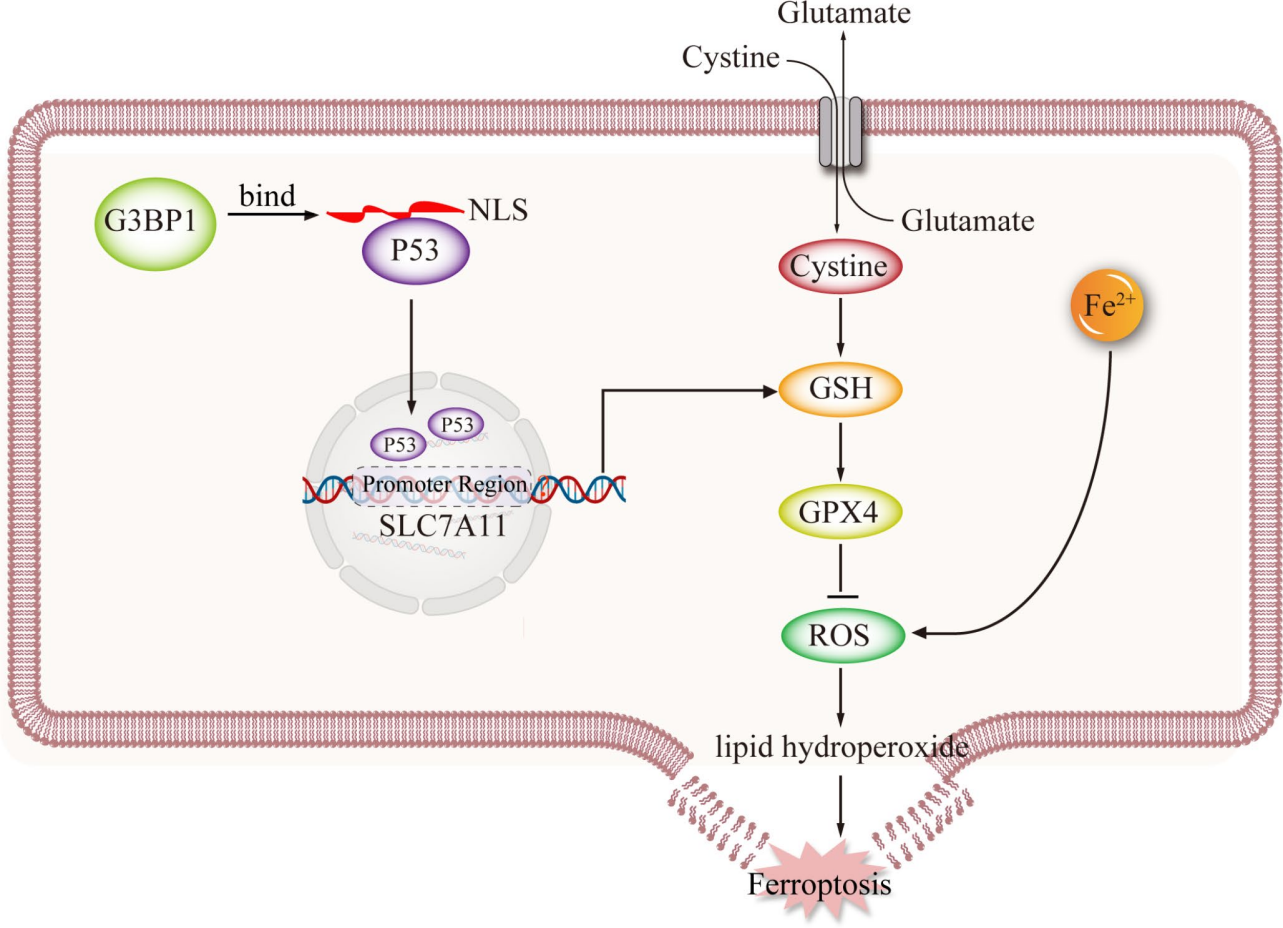
Promoting G3BP1 expression could inhibit P53 entry by binding to the nuclear localization sequence of P53. The inhibition of SLC7A11 transcription was weakened after blocking of P53 binding to the promoter region of the SLC7A11 gene. The SLC7A11-GSH-GPX4 antiferroptotic pathway was subsequently activated, and the level of ferroptosis in ALF hepatocytes was inhibited

In summary, this study was based on previous studies and further explored intervention strategies for ferroptosis in the context of ALF. As shown in Fig. 8, promoting G3BP1 expression could inhibit P53 entry by binding to the nuclear localization sequence of P53. The inhibition of SLC7A11 transcription was weakened after blocking P53 binding to the promoter region of the SLC7A11 gene. The SLC7A11-GSH-GPX4 antiferroptotic pathway was subsequently activated, and the level of ferroptosis in ALF hepatocytes was inhibited. This study was the first to examine the relationship between the G3BP1 protein and the pathogenesis of ALF and contributed to a new and in-depth understanding of the pathogenesis of ALF. These findings suggest that G3BP1 can be upregulated to promote the SLC7A11-GSH-GPX4-ferroptosis signaling pathway to prevent and treat ALF, which provides an innovative and promising idea for clinical practice and has good clinical importance. However, this study still has many limitations. As a key component of SGs, promoting the expression of G3BP1 could induce the production of SGs, which has a protective effect on hepatocytes during ALF [24]. Whether G3BP1 can affect the SLC7A11-GSH-GPX4 antiferroptotic pathway through a SG-dependent pathway is worth further study. Only an adeno-associated virus was used to target G3BP1 in the mouse liver in this study. If possible, we will further use G3BP1^−/−^ transgenic mice in future studies. Moreover, primary hepatocytes can still perform many functions of the liver, such as the decomposition of toxins, urea synthesis, and albumin production. These cells can better restore the development pattern and state of organs in vivo. We will also use primary hepatocytes to model liver organoids in future experimental studies.

More importantly, due to the clinical importance of this study, we confirmed that G3BP1 could regulate the key target molecule P53, which has been proven to be a viable therapeutic target for developing drugs to treat inflammation, immunoregulation and cancer. This study not only focused on reliable therapeutic effects but also opens up a new field of gene-targeted therapy. This study provides a new approach for understanding the pathogenesis and treatment of ALF and lays a foundation for future precision medicine for ALF. Based on this study, a G3BP1 agonist can be used to regulate P53 nuclear translocation and affect ferroptosis in liver parenchymal cells. These findings also provide a scientific basis for the clinical development of drugs to treat ALF.

## Data Availability

All datasets generated for this study are included in the article.
